# Medical Malpractice Lawsuits Involving Trainees in Obstetrics and Gynecology in the USA

**DOI:** 10.3390/healthcare10071328

**Published:** 2022-07-17

**Authors:** Summer Ghaith, Ronna L. Campbell, Jordan R. Pollock, Vanessa E. Torbenson, Rachel A. Lindor

**Affiliations:** 1 Mayo Clinic Alix School of Medicine, Mayo Clinic, Phoenix, AZ 85054, USA; pollock.jordan@mayo.edu; 2 Department of Emergency Medicine, Mayo Clinic, Rochester, MN 55905, USA; campbell.ronna@mayo.edu (R.L.C.); lindor.rachel@mayo.edu (R.A.L.); 3 Department of Obstetrics and Gynecology, Mayo Clinic, Rochester, MN 55905, USA; torbenson.vanessa@mayo.edu

**Keywords:** residents, student/resident supervision, patient safety & quality improvement & value, malpractice, informed consent

## Abstract

Background: While the liability risks for obstetrics and gynecology (ob/gyn) physicians are widely recognized, little is known about how trainees have been involved in ob/gyn lawsuits. Objectives: To characterize involvement of trainees in malpractice lawsuits related to ob/gyn. Methods: The legal database Westlaw was utilized to collect ob/gyn-related malpractice lawsuits involving trainees reported from 1986 to 2020 in the USA. Outcome: Forty-six malpractice cases involving ob/gyn trainees were identified, including 34 cases related to obstetrics and 12 to gynecology. There were 11 cases alleging lack of informed consent, including 7 cases alleging lack of consent for trainee involvement. Of the 34 obstetrics cases, 27 related to procedural complications, 17 to treatment, 13 to diagnosis, and 4 to informed consent. Of these, 17 were decided in favor of the physician, 6 resulted in findings of negligence, 9 had unknown outcomes, and 3 ended in settlement. For the 6 cases ending in a finding of negligence, the mean award was $2,174,472 compared to $685,000 for those that were settled. Of the 12 gynecology cases, 8 related to procedural complications, 7 to informed consent, 3 to diagnosis, and 2 to treatment. Of these, 6 were decided in favor of the physician, 3 resulted in findings of negligence, and 3 had unknown outcomes. For the cases ending in a finding of negligence, the mean award was $465,000. Conclusions and Outlook: This review of malpractice cases highlights types of situations in which trainees are sued and reveals the importance of designing curriculum around faculty training and supervision regarding trainee involvement in patient care.

## 1. Introduction

Obstetrics and gynecology (ob/gyn) remains one of the most frequently sued specialties, with nearly 83% of ob/gyn physicians being sued at least once in their careers [1]. A 2015 survey of ob/gyn physicians revealed that more than 40% experienced at least one claim during training [2]. A study of malpractice cases involving residents from 2001 through 2015 found ob/gyn residents to have the highest number of claims (17%), followed by internal medicine (11%), and surgery (11%). Further, the highest paid-claim rates per 1000 resident-years were in ob/gyn (2.96), followed by neurological surgery (2.01), and plastic surgery (1.43). The highest proportion of catastrophic payments (>$1 million) were also in ob/gyn (18%) [3]. These numbers highlight the importance of better understanding factors that increase ob/gyn trainees’ liability risks.

Studies show that residents and medical students identify liability-related issues as a major factor in considering post-residency work [4,5,6]. However, residency training lacks sufficient medicolegal education, exacerbating trainee fear [7]. Over 50% of practicing ob/gyn physicians report significantly changing their practice out of fear of malpractice lawsuits [2]. This included decreasing high-risk obstetric patient care or discontinuation of obstetrics services altogether. With an estimated shortage of 22,000 ob/gyns in the United States by 2050 [8], it is vital to better understand factors deterring medical students and residents from pursuing careers in ob/gyn. According to a recent study of medical students interested in ob/gyn, 27% ultimately decided on another specialty and cited “fear of being sued” as a major reason [9].

Trainees in the United States are given varying levels of supervision depending on the environment. In general, medical students are generally more closely supervised than residents and fellows, are given progressively more autonomy as they complete additional years of training. Therefore, trainees may not always have someone in the room directly supervising; rather, this may entail only a review of their decision making after a clinical encounter.

The United States legal system is unique on a global scale. Malpractice lawsuits typically arise from acts or omissions that deviate from the standard of care of an average healthcare worker treating a patient under similar circumstances [10]. This paper is specific to the United States, which is thought of as being a highly litigious country and does not necessarily extend to other countries. For example, in the United States, medical malpractice lawsuits are civil actions, whereas in other countries like Italy, there can also be criminal proceedings [11].

While the general liability risks for ob/gyn physicians are widely recognized, little is known about trainee involvement in ob/gyn related lawsuits. An understanding of malpractice lawsuits can help prevent similar situations in the future and allow residency programs to design their curricula to minimize risk for future trainees. Ultimately, knowledge of these cases may decrease the risk of similar negative outcomes and help to highlight optimal care for patients and decrease trainee fear of liability.

The objective of this study was to characterize involvement of trainees in medical malpractice lawsuits related to ob/gyn. We aimed to review the clinical case, patient and legal outcome, and ob/gyn trainee involvement in order to understand why these cases occur.

## 2. Materials and Methods

### 2.1. Study Design and Data Source

We conducted a narrative review of the Thomson Reuters Westlaw Database, a legal database containing lawsuits from federal and state courts in the United States. The legal database contains appellate level cases and select trial court cases and settlements. The database contains tens of thousands of cases, but it is not comprehensive of all legal cases in the United States, as such a database does not exist. A narrative review was chosen in order to provide qualitative information regarding clinical and legal cases and outcomes. Cases and verdicts contain varying levels of clinical detail. This study did not require IRB approval, as the data in this study are publicly available.

### 2.2. Case Selection

**Figure 1:**
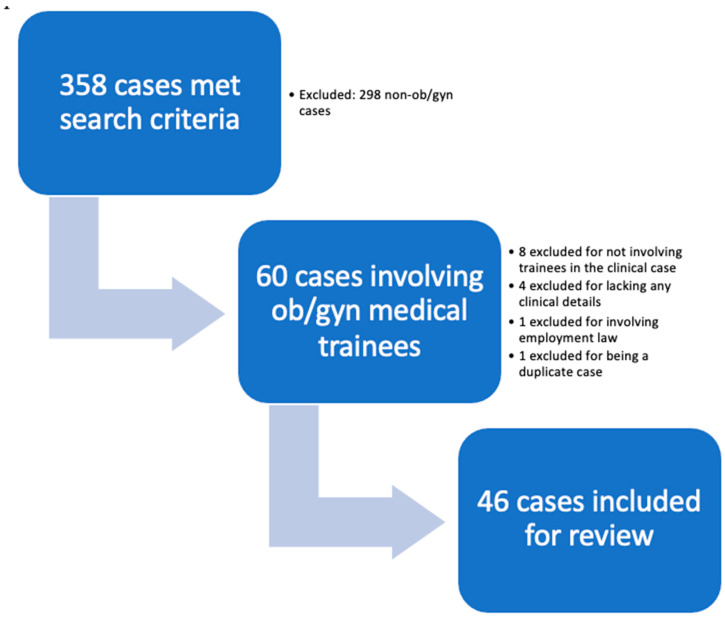
Selection process of cases.

In April 2020, we searched the database for cases involving the terms “malpractice” and one of the following: medical student, student, trainee, PGY, resident, fellow, intern, or postgraduate year, including spelling variations, from 1 January 1914 through 1 January 2020. These search terms ultimately resulted in 358 cases. Authors JRP, RAL, and SG then excluded cases that were not related to ob/gyn. Additionally, cases in which trainees were not involved in any patient care were also excluded. (Figure 1).

### 2.3. Data Collection and Variables

Prior to beginning the case review, the team met to decide on pertinent variables that would be meaningful to characterize ob/gyn malpractice lawsuits involving trainees. Per standardized chart review methodology, a standardized data form was utilized to record abstracted data [12]. The two primary abstractors (RAL and SG) independently reviewed and abstracted data from 5 full cases and met to settle any conflicts. One abstractor (SG) independently reviewed the information for all cases. Ambiguous data were marked and resolved by the senior investigator (RAL).

Variables abstracted included trainee level of education, the state in which the incident occurred, patient demographics, clinical outcomes, categories of alleged error, and legal outcomes, when available.

### 2.4. Statistical Analysis

Categorical data were summarized as frequency of occurrence and continuous data were summarized with means and ranges.

## 3. Results

Among these 46 malpractice cases involving ob/gyn trainees, 44 involved residents (95.6%), two involved fellows (4.4%), and six involved medical students (13.0%). In all of these cases, other team members were named in the lawsuit, in order to prevent a defendant from evading responsibility by attributing fault to someone who was not named in the lawsuit. Thirty-four cases (73.9%) were related to obstetrics and 12 (26.1%) were related to gynecology. Cases were identified throughout 22 states.

## 4. Alleged Error

Seven cases alleged failure to obtain informed consent for involvement of the trainee, including 3 in obstetrics (9%) and 4 in gynecology (33%) (Table 1 and Table 2). Of the 34 obstetrics cases, 27 related to procedural complications (79%), 17 to treatment (50%), 13 to diagnosis (38%), and 1 to informed consent regarding the procedure (3%) (Table 1 and Table 3). Of the 12 gynecology cases, 8 related to procedural complications (67%), 3 to informed consent regarding the procedure (25%), 3 to diagnosis (25%), and 2 related to treatment (17%) (Table 2 and Table 4). Of the 35 cases involving a procedural complication, 33 involved a resident (94%), 4 involved a medical student (11%), and 2 involved a fellow (6%).

## 5. Patient Outcomes

Of the 34 obstetrics cases, 18 resulted in disability of the child (53%), 8 resulted in fetal death (24%), 3 resulted in maternal disability (9%), 2 resulted in loss of fertility (6%), and 4 resulted in maternal death (12%) (Table 1 and Table 3). Of the obstetrics-related injuries, 21 involved delivery complications for the baby (62%), 7 involved prenatal care (21%), 4 were due to delivery complications for the mother (12%), and 2 were due to post-delivery complications of the mother (6%) (Table 1 and Table 3). Of the 12 gynecologic cases, 9 resulted in injury (75%), 1 resulted in permanent disability (8%), 1 resulted in loss of fertility (8%), and 1 resulted in death of the patient (8%) (Table 2 and Table 4).

## 6. Legal Outcomes

Of the 34 obstetrics cases, 17 were decided in favor of the physician (50%), 6 resulted in findings of negligence (18%), 9 had unknown outcomes (26%), and 3 ended in settlement (9%). For the cases ending in a finding of negligence, the mean award amount was $2,174,472 compared to $685,000 for those that were settled (Table 1 and Table 3).

Of the 12 gynecology cases, 6 (50%) were decided in favor of the physician, 3 (25%) resulted in findings of negligence, 3 (25%) had unknown outcomes, and none ended in settlement. For the cases ending in a finding of negligence, the mean award amount was $465,000 (Table 2 and Table 4).

The mean time from incident to lawsuit resolution and report for obstetrics cases was 6.9 years (range, 3–25 years). The mean time for cases that found the resident not liable was 7.1 years (range, 3–25 years), compared to 6.6 years (range 5–9) for cases decided against the physicians, and 6.0 years (range, 3–9 years) for cases that ended in settlement.

The mean time from incident to lawsuit resolution and report for gynecology cases was 6.6 years (range, 3–10 years). The mean time for cases that found the resident not liable was 7.1 years (range, 4–10 years), compared to 7.5 years (range 5–10) for cases decided against the physicians.

## 7. Discussion

We identified 46 cases involving trainees in ob/gyn from 1986 through 2020. We found residents were the most likely trainees to be involved in a lawsuit and more cases involved obstetrics than gynecology. Additionally, malpractice cases were often due to procedural complications or problems of informed consent. In this study, approximately 1 in 4 cases resulted in settlement or a finding of liability against the physicians.

Cases took an average of 7 years from incident to resolution, even for cases in which those involved were ultimately found to have no liability. This is several years longer than other studies reporting an average time of 4 years for case resolution [13,14,15]. Physicians involved in a lawsuit are more likely to experience worse health-related quality of life, including general health, mental health, and vitality [16]. This psychological toll increases risk for burnout in ob/gyn physicians who already have high rates of burnout including feelings of depersonalization and emotional exhaustion [17]. This highlights the heavy toll that lawsuits can have on those involved regardless of the legal outcome due to the long length of an open case.

The majority of lawsuits in this study revolved around procedural errors, in both obstetrics and gynecology. Almost all of the gynecology cases involved patient injuries from procedural errors, such as bowel perforations and ureteral injuries. Within obstetrics, the most common procedure leading to a lawsuit was childbirth, specifically in cases in which the infant had a bad outcome, such as brachial plexus injury or a permanent neurologic deficit. While trainees surely benefit from being involved in high-risk procedures and need to learn the skills required in these situations, programs also need to ensure that they are being thoughtful about ensuring adequate supervision and documentation of appropriate level of training.

A second major theme in lawsuits emerging from this study was failure to obtain informed consent. We found that 16% of cases were due to alleged failure to obtain informed consent for the trainee’s role in the procedure or delivery. The Centers for Medicare and Medicaid Services, as a condition of participation, requires (except in an emergency) that the informed consent process for surgeries includes a discussion about the fact that trainees will be involved, including their anticipated roles and the level of supervision provided by the attending physician [18]. While these points are only required by CMS for discussion and not necessarily as part of the signed form, including robust documentation of these discussions, with specific mention of trainee involvement, could potentially prevent future lawsuits.

The major limitation of this study is reliance on a legal database. First, the database is not a comprehensive collection of cases. Rather, it focuses primarily on cases that were appealed and those chosen to be published by court reporters. Nevertheless, our study is not meant to be a quantitative study, rather, it provides a descriptive review and analysis of malpractice cases involving medical trainees in obstetrics and gynecology. Second, because the primary focus of these cases is the legal outcome, they often did not include pertinent medical information. Despite this limitation, we believe the cases included in our study provide valuable insight into malpractice cases of trainees in obstetrics and gynecology and provide a framework for further study.

## 8. Conclusions

Residents were the most likely trainees to be involved in a lawsuit, and obstetrics cases were more common than gynecology cases in our study. Procedural complications and lack of informed consent were the most common alleged errors in these cases. This review of malpractice cases highlights types of situations in which trainees are sued and reveals the importance of designing curriculum around faculty training and supervision regarding trainee involvement in patient care. Encouraging communication between trainees and attending physicians can reduce the number of lawsuits against trainees. A better understanding of these trends can help programs minimize trainees’ future liability risks, promoting trainee well-being and limiting stress and potential burnout related to liability. Additionally, this understanding can help programs provide optimal patient care.

## Figures and Tables

**Table 1:** Characteristics of 34 legal cases involving trainees in obstetrics.

**Injuries**	**N (%)**
Delivery complications for fetus	21 (62%)
Inadequate prenatal care	7 (21%)
Delivery complications for mother	4 (12%)
Post-delivery complications for mother	2 (6%)
**Patient outcome**	
Permanent disability (fetus)	18 (53%)
Death (fetus)	8 (24%)
Permanent disability (mother)	3 (9%)
Infertility (mother)	2 (6%)
Death (mother)	4 (12%)
**Category of alleged error**	
Procedural complication	27 (79%)
Treatment	17 (50%)
Diagnosis	13 (38%)
Informed consent of trainee involvement	3 (9%)
General Informed consent	1 (3%)
**Outcome of lawsuit**	
No liability	16 (47%)
Unknown	9 (26%)
Negligence	6 (18%)
Settlement	3 (9%)
**Amount of settlement/judgement**	**Mean (range)**
Cases ending in finding of negligence	$2,174,472 ($457,250–$7,250,000)
Cases ending in settlement	$685,000 (only 1 was known)

**Table 2:** Characteristics of 12 legal cases involving trainees in gynecology.

**Patient Outcome**	**N (%)**
Injury	9 (75%)
Permanent disability	1 (8%)
Infertility	1 (8%)
Death	1 (8%)
**Category of alleged error**	
Procedural complication	8 (67%)
Informed consent of trainee involvement	4 (33%)
General Informed consent	3 (25%)
Diagnosis	3 (25%)
Treatment	2 (17%)
**Outcome of lawsuit**	
No liability	6 (50%)
Negligence	3 (25%)
Unknown	3 (25%)
Settlement	0 (0%)
**Amount of settlement/judgement**	**Mean (range)**
Cases ending in finding of negligence	$465,000 ($170,000–$700,000)
Cases ending in settlement	N/A

**Table 3:** Summary table of individual legal cases involving trainees in obstetrics.

Year of Report	Trainee Level Involved	Location	Patient Outcome—Mother	Patient Outcome—Fetus	Legal Outcome ($ amount)	Category of Alleged Error	Alleged Error (Partum Phase)
1983	Medical student; resident	Louisiana	-	Cognitive delay, brain damaged, spastic quadriplegia, deaf, and almost totally blind	No Liability	procedural complication; diagnosis; informed consent for vaginal procedure	ante, intra
1992	Resident	Missouri	Death	-	No liability	procedural complication; treatment	ante, intra, post
1993	Resident	California	-	Right arm Erb’s palsy	Negligence ($685,000)	procedural complication; diagnosis; treatment	ante, intra
1993	Resident	Ohio	-	Death	Unknown	procedural complication	pre, intra
1996	Resident	North Carolina	-	Cognitive delay; cerebral palsy, severe spastic quadriparesis, seizures	Unknown	procedural complication	ante, intra, post
1996	Resident	Virginia	-	Spinal injury, permanent paralyzation	Unknown	procedural complication	ante, intra
1997	Medical student	Illinois	-	Upper brachial plexus injury and Erb’s palsy	Negligence ($1,000,000)	procedural complication	intra
1999	Medical student; resident	Illinois	Gangrene requiring amputation of multiple extremities	-	Negligence (7,250,000)	diagnosis; treatment	ante, intra
2000	Resident	Alabama	-	Seizure disorder and hemiplegia	Unknown	procedural complication	ante, intra
2001	Resident; fellow	Kentucky	-	Brain damage	No liability	procedural complication	ante, intra
2001	Resident	Massachusetts	Permanent neurologic deficit	-	No liability	informed consent; treatment	ante, intra
2001	Resident	New York	-	Patient miscarriage	Unknown	diagnosis; treatment	ante
2001	Resident; fellow	Kentucky	-	Brain damage	No liability	procedural complication	ante, intra
2002	Resident	Virginia	-	Brain trauma	No liability	procedural complication	intra
2005	Resident	Texas	Emergency hysterectomy; infertility	Fetal death > 20 weeks	No liability	procedural complication	intra, post
2006	Resident	Kansas	-	Brachial plexus injury	No liability	procedural complication	intra
2007	Resident	Massachusetts	-	Right Erb’s palsy and loss of function in right arm	No liability	procedural complication; diagnosis; treatment; informed consent	intra
2007	Resident	Pennsylvania	-	Brachial plexus injury	Negligence ($457,250)	treatment; procedural complication	ante, intra
2007	Resident	Michigan	-	Neurological deficits (cerebral palsy, cognitive delays and developmental delays)	Settlement	procedural complication; diagnosis; treatment; informed consent	intra, post
2007	Resident	California	-	Severe brain damage, developmental delay, collapsed left lung, gastrostomy, tracheostomy	Settlement ($5,300,000)	procedural complication	ante, intra
2008	Resident	Illinois	-	Cerebral palsy and physical impairment and cognitive damage	No liability	procedural complication; diagnosis; treatment	ante
2008	Resident	Michigan	Pubic symphysis diastasis leading to permanent nerve damage and infertility	-	No liability	diagnosis; treatment	ante, intra
2009	Resident	Massachusetts	Death from a post-operative intra-abdominal abscess	-	No liability	diagnosis; treatment	post-op
2009	Resident	Wisconsin	-	Death of 1 of 2 twins	Unknown	diagnosis; treatment	ante
2010	Resident	Virginia	-	Death	No liability	procedural complication	pre, intra
2011	Resident	Pennsylvania	-	Death	Negligence ($2,154,583)	procedural complication; diagnosis; treatment	ante, intra
2011	Resident	Texas	-	Injury brachial plexus	Unknown	procedural complication	intra
2012	Resident	Ohio	-	Fetal death > 20 weeks	No liability	procedural complication; diagnosis; treatment	ante, intra
2013	Resident	Kentucky	-	Cerebral palsy	No liability	procedural complication	ante, intra
2014	Resident	Texas	Death of patient	Death of two children	Unknown	treatment; medication	ante, intra
2015	Medical student	Missouri	-	Brachial plexus injury	Settlement	procedural complication	intra
2016	Resident	Nevada	Uterine/ureteral damage	-	No liability	procedural complication	procedural
2016	Resident	California	-	Hemiplegic cerebral palsy secondary to stroke	Unknown	procedural complication	ante, intra
2017	Resident	Pennsylvania	Death	-	No liability	procedural complication; treatment	pre, intra, post

**Table 4:** Summary table of individual legal cases involving trainees in gynecology.

Year of Report.	Trainee Level Involved	Location	Patient Outcome	Legal Outcome ($ Amount)	Category of Alleged Error	Alleged Error
1992	Medical student; residents	Washington DC.	Surgical removal of uterus, fallopian tubes, and ovaries (infertility, migraine headaches, depression)	Negligence ($170,000)	informed consent	procedural
1998	Resident	Tennessee	Pregnancy after a tubal ligation and sterilization	No liability	procedural complication	procedural
2000	Resident	Mississippi	Perforated bowel	Negligence ($525,000)	informed consent; procedural complication	procedural; post-procedural
2002	Resident	Pennsylvania	Complications after surgery; respiratory distress	No liability	informed consent; diagnosis; treatment	procedural
2002.	Resident	Ohio	Damaged ureter	Unknown	procedural complication; informed consent	procedural
2006	Resident	Colorado	Left ureteral obstruction, flank pain and swollen kidney	No liability	procedural complication; informed consent	procedural
2007	Resident	Illinois	Death due to ARDS and bowel perforation	No liability	procedural complication; diagnosis	procedural
2008	Resident	Indiana	Sciatic nerve damage	Negligence ($700,000)	procedural complication	procedural
2014	Resident	Indiana	Wound drainage and swelling causing further surgery	No liability	informed consent	procedural
2014	Resident	Texas	Perforated bowel	Unknown	informed consent; procedural complication; diagnosis; treatment	procedural, post-procedural
2019	Resident	Louisiana	Vaginal injuries	No liability	procedural complication	procedural
2019	Medical student	Massachusetts	Sciatic nerve injury	Unknown	procedural complication	procedural

## Data Availability

All data were abstracted from Westlaw, an online legal database.
